# The distribution and adipogenic potential of perivascular adipose tissue adipocyte progenitors is dependent on sexual dimorphism and vessel location

**DOI:** 10.14814/phy2.12993

**Published:** 2016-10-12

**Authors:** G. Andres Contreras, Kyan Thelen, Nadia Ayala‐Lopez, Stephanie W. Watts

**Affiliations:** ^1^ Department of Large Animal Clinical SciencesMichigan State UniversityEast LansingMichigan; ^2^ Department of Pharmacology and ToxicologyMichigan State UniversityEast LansingMichigan

**Keywords:** Adipocyte progenitors, adipogenesis, perivascular adipose tissue

## Abstract

There are sex associated differences in the risk for cardiovascular comorbidities in obesity and metabolic syndrome. A common clinical finding in these diseases is the expansion of perivascular adipose tissues (PVAT) which is associated with alterations in their role as regulators of vessel function. PVAT hyperplasia and hypertrophy are dependent on the biology of populations of adipocyte progenitor cells (APC). It is currently unclear if PVAT enlargement diverges between males and females and the mechanisms linking APC biology with sexual dimorphism remain poorly understood. This study tested the hypothesis that vessel location and sexual dimorphism affect the distribution and adipogenic capacity of APC in cardiovascular disease risk relevant PVAT sites. PVAT from thoracic aorta (aPVAT) and mesenteric resistance arteries (mPVAT) was collected from 10‐week‐old female and male Sprague–Dawley rats. Differences in APC distribution in stromal vascular fraction cells from PVAT were determined. APC were defined as cells expressing CD34, CD44, and platelet derived growth factor *α*. In both sexes aPVAT had fewer APC compared to mPVAT and perigonadal adipose tissue (GON). Sex‐related differences were observed in the expression of CD34, where females had fewer CD34^+^ cells in PVATs. APC proliferation and adipogenic capacity in vitro were not affected by sex. However, APC from aPVAT had a lower proliferation capacity compared to mPVAT. These data demonstrate that the distribution of APC within PVAT exhibits sexual dimorphism and is affected by anatomical location.

## Introduction

The risk for cardiovascular diseases (CVD) in conjunction with obesity and metabolic syndrome exhibits sex‐related differences. For example, hypertension has a greater prevalence in obese men compared to obese premenopausal women (Lima et al. 2012). Higher blood pressure in males is also observed in rat models of diet‐induced obesity (Coatmellec‐Taglioni et al. 2002). The development of hypertension during obesity is directly associated with the expansion of perivascular adipose tissues (PVAT), major paracrine regulators of blood vessel function (Watts et al. 2013), through hyperplasia and hypertrophy (Brown et al. 2014). In males and females, PVAT adipocyte phenotype varies depending on its anatomical location. Thoracic aorta PVAT (aPVAT) is composed of mostly multilocular adipocytes (brown) that secrete the thermogenic uncoupling protein‐1 (UCP1) and contain fewer unilocular cells (Fernández‐Alfonso et al. 2010; Watts et al. 2011). In contrast, adipocytes in PVAT surrounding mesenteric resistance arteries (mPVAT) are mostly unilocular (white) with some brown adipocyte areas possibly resembling inducible brown (brite) adipocytes (Watts et al. 2011).

The expansion of aPVAT and visceral adipose, where mPVAT is found, shows sexual dimorphism in rodents and humans during obesity and aging. For example, women have a higher prevalence for larger aPVAT compared to age‐matched men (Britton et al. 2012). Although not described in mPVAT specifically, lean and obese men have larger mesenteric fat thickness and adipocyte size compared to women (Fried and Kral 1986; Borruel et al. 2013). Whereas sexual dimorphism influences PVAT expansion, it is currently unknown if differences in the populations of adipocyte progenitor cells (APC) are part of the mechanisms leading to these sex‐related differences in PVAT.

Adipose tissue hyperplasia and hypertrophy reflect the phenotype of APC (Police et al. 2009; Dodson et al. 2014). These cells reside in the nonbuoyant cellular fraction of adipose tissues, the stromal vascular fraction (SVF), and are usually characterized by the expression of certain cell surface markers. Although a definitive APC marker profile has not been established (Cawthorn et al. 2012), recently, a unique set of APC exhibiting cell surface proteins CD34 and platelet‐derived growth factor receptor alpha (PDGFR*α*) were characterized by two independent groups (Lee et al. 2012; Berry and Rodeheffer 2013). These APC were identified in diverse adipose depots including interscapular brown (BAT), perigonadal (GON), and inguinal subcutaneous fat using lineage tracing. CD34^+^ PDGFR*α* ^+^ cells have high adipogenic potential and can express multilocular or unilocular phenotypes after adipogenesis depending on anatomical location and metabolic status (Lee et al. 2013).

Studying specific APC subpopulations in each PVAT site facilitates the evaluation of inherent physiognomies that may be affected by sexual dimorphism and could result in unique responses to adipogenic stimuli such as APC proliferation and/or adipocyte hypertrophy (Joe et al. 2009). Remarkably, APC‐specific characteristics are retained and maintained even when cultured and were described for subcutaneous and nonperivascular visceral fat pads (Tchkonia et al. 2006; Macotela et al. 2012). However, despite the importance of PVAT in the pathogenesis of CVD, sex‐related differences in the expansion capacity of aPVAT and mPVAT remain poorly understood and the specific phenotype of APC in PVAT is unknown. In this study, we hypothesized that anatomical location and sexual dimorphism affect the distribution and adipogenic capacity of APC in PVAT.

## Material and Methods

### Animals

Female and male Sprague–Dawley rats (8–10 weeks of age, females 230–250 g, males 250–300 g; Charles River Laboratories, Inc., Portage, MI) were maintained on a normal chow diet containing 4.3% fat, 19.2% protein, and 67.3% carbohydrate as % of dry weight (cat # D12450J; Research Diets, New Brunswick, NJ). Rats were euthanized by an intraperitoneal injection of 60–80 mg/kg of pentobarbital. All animal procedures were approved by the Michigan State University Animal Care and Use Committee Procedures. All guidelines are in accordance with the “The Guide for the Care and Use of Laboratory Animals” (Garber et al. 2011).

### Tissue processing and immunohistochemistry

Tissue samples were fixed with 4% formalin, embedded in paraffin, and cut into 5‐*μ*m‐thick sections. Low‐magnification panoramic images were assembled using Image Composite Editor (Microsoft Research, Redmond, WA). Adipocyte cell sizes were determined in hematoxylin‐ and eosin‐stained paraffin sections by measuring the area of >100 adjacent cells from five randomly selected fields per section using ImageJ software (National Institutes of Health, Bethesda, MD). Frequency distribution was determined and analyzed using GraphPad Software (GraphPad, San Diego, CA). For immunostaining, specimens were fixed in 10% neutral‐buffered formalin followed by processing, embedding in paraffin and sectioning on a rotary microtome at 4–5 *μ*m. Sections were placed on charged slides and dried at 56°C overnight. The slides were subsequently deparaffinized in xylene and hydrated through descending grades of ethyl alcohol to distilled water. Slides were placed in Tris‐buffered saline (TBS) pH 7.4 (Scytek Labs, Logan, UT) for 5 min for pH adjustment. Following TBS, Heat Induced Epitope Retrieval in Citrate Plus pH 6.0 (Scytek Labs) was performed in a vegetable steamer at 100°C for 30 min; followed by a 10‐min room temperature incubation and rinses in several changes of distilled water. Following pretreatment, standard Micropolymer staining steps were performed at room temperature on the Biocare intelliPATH automated staining instrument (Biocare, Concord, CA). All staining steps were followed by rinses in TBS Autowash Buffer + surfactant (Biocare). After blocking for nonspecific protein with Background Punisher (Biocare) for 5 min, sections were incubated with primary antibody for 30 min with a polyclonal rabbit anti‐mouse UCP1 antibody @ 1:800 (Alpha Diagnostics International, San Antonio, TX) in Normal Antibody Diluent (NAD; Scytek). Rabbit on Rodent HRP – Polymer (Biocare) was added and incubated for 30 min. Reaction development utilized a Biocare AEC chromogen incubation of 5 min followed by counterstain in Gill 2 Hematoxylin (Cancer Diagnostics, Durham, NC) for 5 sec followed by dehydration, clearing, and mounting with synthetic mounting media.

### Gene expression analysis

Adipose tissue RNA was extracted in Trizol (Thermo Scientific, Waltham, MA) and purified with RNeasy (Qiagen, Valencia, CA) columns. Conversion to cDNA was performed using the Applied Biosystems High Capacity cDNA Archive Kit (Applied Biosystems, Foster City, CA). qPCR was performed using SYBR (ABsolute Blue QPCR SYBR; Thermo Scientific). Samples were assayed in triplicate with 714 pg/*μ*L of cDNA on a single run of the Wafergen Smartchip System (WaferGen Biosystems, Fremont, CA). Primer descriptions are included in Table 1. A nonreverse transcriptase control was run to ensure that genomic DNA was not being amplified. Expression data were normalized to the housekeeping gene *Rps29*, the average C_T_ across adipose sites was 21.59 ± 0.1 per 40 cycles.

**Table 1:** Primers for PCR

Gene	GenBank	Sequence (5′–3′)
*Adipoq*	NM_144744	Forward: CCGTGATGGCAGAGATGG
		Reverse: CTCCTGTCATTCCAGCATCTC
*Cebpa*	NM_007678	Forward: CTGCGAGCACGAGACGTCTATAG
		Reverse: TCCCGGGTAGTCAAAGTCACC
*Dio2*	NM_031720	Forward: ATGGGACTCCTCAGCGTAGA
		Reverse: GCACAGGCAAAGTCAAGAAG
*Pgc1α*	XM_001362678	Forward: ACCCACAGGATCAGAACAAACC
		Reverse: GACAAATGCTCTTTGCTTTATTGC
*Plin1*	NM_013094	Forward: GTAGAATATCTCCTGCCACCA
		Reverse: TGTGTCGAGAAAGAGTGTTGG
*Pparg*	NG_011749	Forward: GGTGTGATCTTAACTGTCGG
		Reverse: TTCAGCTGGTCGATATCACT
*Prdm16*	NM027504	Forward: TGATGGCCGCTTGGAAGA
		Reverse: TCACTGCCATCCGACATGTC
*Rps29*	X59051	Forward: GCCAGGGTTCTCGCTCTTG
		Reverse: GGCACATGTTCAGCCCGTAT
*Tbx1*	NM_001108322	Forward: ATGGGACGAGTTCAATCAGC
		Reverse: GAGCATGTAGTCAGCCATCG
*Tcf21*	NM_001032397	Forward: GTTACATTCACCCAGTCAACCT
		Reverse: CCAGACTCACACCTCCAAG
*Tmem26*	NM_001107623	Forward: ATGCTCCAGTTTCCCCTTG
		Reverse: CCATAGATCCGCACTGTACTG
*Ucp1*	NC_005118.3	Forward: CTTCTCAGCCGGCGTTTCTG
		Reverse: GGTGATGGTCCCTAAGACACC
*Zic1*	NM_022677	Forward: TTTCCCTGCCCGTTTCC
		Reverse: CCTCGAACTCGCACTTGA

### Adipocyte progenitor isolation and flow cytometry

Adipocyte progenitor cells were harvested from perigonadal (GON), mPVAT, and aPVAT. Fat pads were placed on ice in Krebs Ringer Bicarbonate Buffered Solution (KRBB) with HEPES 10 mmol/L (pH = 7.4). Tissues were minced (1–3 mm pieces) and digested in collagenase type I solution (1 mg/mL; Worthington Biochemical, Lakewood, NJ) in KRBB containing 4% BSA in 1.5‐mL microcentrifuge tubes. Samples in collagenase solution were incubated at 37°C in a rotisserie incubator for 1 h. Digested material was sequentially filtered through 100, and 40 *μ*m cell strainers (Corning, Corning, NY). Resulting filtrate was centrifuged at 4°C for 10 min at 300 × *g*. Pellets containing the SVF cells were resuspended in erythrocyte lysis buffer solution (5 mL) containing 154 mmol/L ammonium chloride, 10 mmol/L potassium bicarbonate, and 0.1 mmol/L EDTA and rocked on a platform for 5 min at room temperature. SVF suspensions were centrifuged at 4°C for 10 min at 300 × *g* and then resuspended in fluorescence‐activated cell sorting (FACS) solution containing PBS, 10% sodium azide solution, 2% fetal bovine serum, and 0.5 mmol/L EDTA for flow cytometry analysis.

For flow cytometric analysis, cell suspensions were incubated with antibodies against cell surface antigens: rabbit anti‐human PDGFR*α* (PAS 17623; Thermo Scientific), mouse anti‐human CD34‐PEC7 (sc‐7324; Santa Cruz Biotechnology, Dallas, TX), rat anti‐mouse CD44 (822201; BioLegend, San Diego, CA). After a 30‐min incubation at 4°C, cells were washed three times with FACS solution. Next, SVF cells were incubated for 30 min at 4°C with the corresponding secondary antibody that included either donkey anti‐rabbit IgG‐DyLight 405 (711‐475‐152; Jackson ImmunoResearch, West Grove, PA) or donkey anti‐rat IgG (712‐165‐153; Jackson ImmunoResearch). After secondary antibody exposure, cells were washed with FACS solution three times. Finally, SVF cells were fixed with 2% formaldehyde solution and stored out of direct light for up to 3 days. Flow cytometry analysis was performed using the FACSdiva software on an Influx Sorter Flow Cytometer (BD Biosciences, San Jose, CA).

For culture and adipogenesis assays, APC were isolated from SVF based on CD34 and PDGFR*α* cell surface expression. First, SVF were incubated with FITC‐conjugated anti‐CD34 (sc‐7324; Santa Cruz Biotechnology) and then with anti‐FITC MultiSort MicroBeads (Anti‐FITC MultiSort Kit; Miltenyi Biotec, San Diego, CA) to separate CD34^+^ and CD34^−^ cells. After release of anti‐FITC MultiSort MicroBeads, the CD34^+^ fraction was incubated with anti‐PDGFR*α* (PAS 17623; Thermo Scientific), and PDGFR*α* ^+^ cells were isolated with anti‐rabbit IgG1 MicroBeads (Miltenyi Biotec). All steps of the APC isolation were performed at 4°C.

### Cell culture and adipogenesis induction

After isolation, the SVF and APC were cultured in basal media containing Dulbecco's Modified Eagles Medium (DMEM): F12 (Corning), 15% fetal calf serum (Corning), antibiotic/antimycotic mixture (Corning), 100 *μ*mol/L ascorbic acid (Sigma‐Aldrich, St Louis, MO), 33 *μ*mol/L biotin (Sigma‐Aldrich), 17 *μ*mol/L pantothenate (Sigma‐Aldrich), 2 mmol/L l‐glutamine (Corning), and 20 mmol/L HEPES (Corning) with replacement every 2 days. Plastic adherent SVF were used after three serial passages. For APCs, the cells were supplemented with growth factors as described previously including (Macotela et al. 2012): epidermal growth factor (10 ng/mL, cat. 400‐25, PeproTech, Rocky Hill, NJ), leukemia inhibitory factor (10 ng/mL, cat. 250‐02; PeproTech), platelet‐derived growth factor BB (10 ng/mL, cat. CYT‐740; Prospec Bio, East Brunswick, NJ), and basic fibroblast growth factor (5 ng/mL, cat. 450‐33; PeproTech). SVF and APCs were plated at 50,000 cells/well in 24 well plates and 10,000 cells/well in 48 well plates for adipogenesis assays. To analyze proliferation capacity of SVC and APC, these cells were seeded in black 96 well plates at 1 × 10^2^ cells/well and cell proliferation was evaluated using the nonradioactive CyQUANT^®^ Cell Proliferation assay (Thermo Scientific) at 8, 24, 48, and 96 h.

Stromal vascular fraction and APC were induced to differentiate after 48 h at 100% confluency (day 0) using a basal media formulation supplemented with 10% fetal bovine serum, 2.5 *μ*g/mL insulin (Corning), and the following reagents from Sigma‐Aldrich: 0.5 mmol/L 2‐isobutyl‐1‐methylaxanthine (IBMX), 1 *μ*mol/L dexamethasone, and 200 pmol/L T3. Thereafter, cells were maintained in media without IBMX and dexamethasone, for 14 days with media changes every 48 h. For APCs, basal media were supplemented for 48 h postconfluency and prior to induction with bone morphogenic protein 4 (3.3 nmol/L, cat. CYT‐081; ProspecBio) as indicated in (Macotela et al., 2012) and then induced to differentiate similar to the SVF. Lipid accumulation was assessed quantitatively using the AdipoRed^™^ assay (Lonza, Allendale, NJ) in an Infinite M1000 Pro Tecan microplate reader (Tecan, Männedorf, Switzerland) and by Oil Red O staining with imaging performed on a Nikon Eclipse inverted microscope equipped with a Nikon Digital Sight DS‐Qil camera (Nikon Group, Otowara, Japan).

### Statistical analysis

Data are reported as means ± SEM. Data were analyzed by one‐ or two‐way ANOVA using GraphPad Software (GraphPad) or Proc GLM (SAS, Cary, NC). Post hoc comparisons were performed using Tukey's test. Statistical significance was set at *P* ≤ 0.05.

## Results

### Sex and vessel location influence PVAT adipocyte morphology

**Figure 1:**
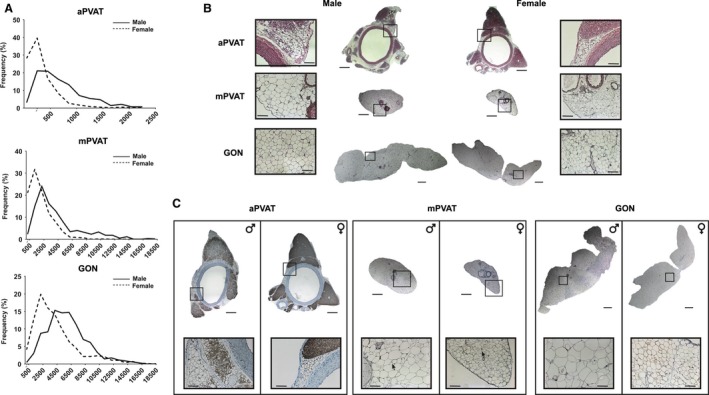
Perivascular adipose tissue (PVAT) adipocyte phenotype varies depending on sex and anatomical location in 10‐week‐old Sprague–Dawley rats. (A) Distribution of white adipocyte areas in aortic (aPVAT) and mesenteric (mPVAT) PVAT, and perigonadal fat (GON), *n* = 6. Representative low and high‐magnification images of aPVAT, mPVAT, and GON stained with hematoxylin and eosin (B) and with antibodies against UCP1 (C) from male and female rats. Scale bars = 0.22 mm (low magnification) and 110 *μ*m (high magnification). Arrows indicate adipocytes exhibiting UCP1 staining in mPVAT.

For basic characterization of the morphology of PVAT adipocytes depending on anatomical site, imaging of hematoxylin–eosin‐stained sections from 10‐week‐old male and female rats was performed. In these animals, aPVAT exhibited well defined areas of adipocytes with brown and white phenotypes. Regions with brown adipose morphology were predominant and their adipocytes had multiple small lipid droplets and intense basophilic staining. White adipocyte areas were localized on the periphery of aPVAT. These unilocular cells were smaller in area compared to white adipocytes from other pads (Fig. 1A and B). Evaluation of aPVAT adipocyte area distribution indicated that females had smaller white adipocytes with a higher percentage of cells with areas 250–500 *μ*m^2^ compared to males (*P *<* *0.001, chi square test, Fig. 1A). Reflecting a predominant brown phenotype, the majority of aPVAT adipocytes from males and females were intensely stained for UCP1 protein while few cells exhibited signals of this brown phenotype marker in mPVAT and no staining was observed in GON (Fig. 1C). In contrast to aPVAT, the majority of adipocytes from mPVAT were white adipocytes with a single lipid droplet and larger cell area than aPVAT white adipocytes, although some multilocular UCP1‐positive adipocytes were observed (Fig. 1A–C). Sex differences were observed in the area distribution of white mPVAT adipocytes, over 30% of mPVAT adipocytes from females had areas >1000 and <2000 *μ*m^2^ compared to less than 20% in males (*P *<* *0.001, Fig. 1A). Conversely, males had higher number of larger (>6000 *μ*m^2^) fat cells. Compared to white adipocytes in PVATs, adipocytes from nonperivascular white adipose pads (GON) exhibited significantly larger cellular areas (Fig. 1A and B). Females had smaller GON adipocytes with a higher percentage of fat cells with areas <4500 *μ*m^2^ compared to males (*P *<* *0.001, Fig. 1A).

### Sex influences APC populations distribution

**Figure 2:**
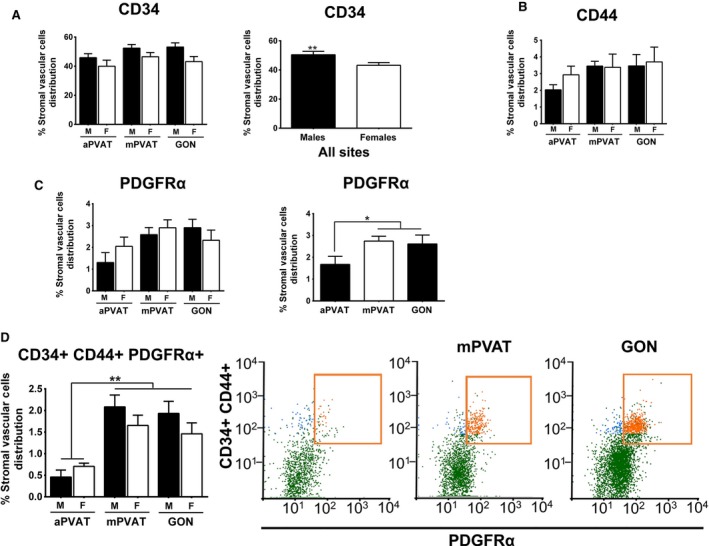
Adipocyte progenitor cell (APC) population distribution in aortic (aPVAT) and mesenteric (mPVAT) perivascular adipose tissues and perigonadal (GON) fat depot is affected by sex and anatomical location in Sprague–Dawley rats. Quantification of cell surface CD34 (A), CD44 (B), and platelet derived growth factor alpha (PDGFR*α*) (C). (D) Cells co‐expressing APC cell surface markers CD34, CD44, and PDFGR*α* are identified in orange and located within square box of the flow cytometry output. Values are percentage of the SVF population ± SEM as measured by flow cytometry (*n* = 5). Significant differences indicated by * (*P* < 0.05) and ** (*P* < 0.01).

Adipocyte progenitor cells were characterized in SVF cells harvested from aPVAT, mPVAT, and GON from 10‐week‐old male and female rats. Quantification of cells expressing surface markers was performed using flow cytometry (Fig. 2). APC were defined as cells coexpressing CD34, CD44, and PDGFR*α*, as previously reported (Lee et al. 2012). When evaluating the expression of individual markers in SVF cells from all sites, males had a higher number of SVF cells expressing CD34 compared to females, but no differences were observed in the number of CD44^+^ and PDGFR*α* ^+^ between sexes (Fig. 2A–C). Assessment of PDGFR*α* cell surface expression in SVF cells by site demonstrated that fewer aPVAT cells were positive for this marker compared to mPVAT and GON (Fig. 2C). When evaluating coexpression of CD34, CD44, and PDGFR*α*, the numbers of APC were significantly lower in aPVAT compared to mPVAT and GON (Fig. 2D).

### Sex and site affect PVAT adipocyte gene signature patterns

**Figure 3:**
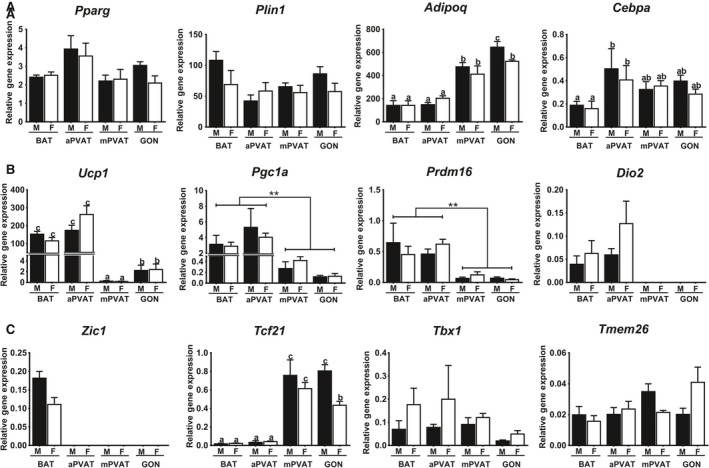
Perivascular adipose tissues exhibit diverse gene signature patterns. Aortic (aPVAT) and mesenteric (mPVAT) perivascular adipose tissues and brown (BAT) and perigonadal (GON) fat depots were collected from 10‐week‐old males and females fed a standard chow diet. Gene expression of adipogenic (A), brown adipocyte markers (B), and adipose identity markers (C), was evaluated by RT qPCR and normalized to *Rps29*. Data are means ± SEM (*n* = 6). Significant differences are indicated by * and letters a, b, c (*P* < 0.05), ** (*P* < 0.01).

To characterize the gene signature of aPVAT and mPVAT, the expression of adipogenic and recently validated brown (*Zic1*), white (*Tcf21*), and brite (*Tbx1, Tmem26*) adipocyte marker genes (de Jong et al. 2015) was evaluated using RT‐qPCR in 10‐week‐old males and females fed standard chow. Gene expression in PVATs was compared to that of BAT and white (GON) adipose depots. Among sites and sexes, no differences were observed in the expression of *Pparg*, a regulator of adipogenesis, and *Plin1*, encoding a lipid droplet associated protein. Site differences were observed in the expression of the gene encoding adiponectin (*Adipoq*), which had higher expression in the mostly white depots, mPVAT and GON, compared to the brown sites, aPVAT and BAT (Fig. 3A). Remarkably, the expression of *Adipoq* was significantly higher in males’ GON tissue compared to that in females. aPVAT had higher transcription levels of the adipogenic marker *Cebpa* compared to BAT. However, aPVAT expression of *Cebpa* did not differ from that in mPVAT or GON.

As expected, BAT and aPVAT exhibited higher expression of brown adipocyte‐related genes including *Ucp1*,* Pgc1a*, and *Pdrm16* compared to mPVAT and GON (Fig. 3B). In mPVAT and GON, *Dio2* was not detected. Expression of *Zic1*, a brown adipocyte specific marker, was only detected in BAT (Fig. 3C). In contrast, *Tcf21* expression was high in mPVAT and GON reflecting the predominantly white adipocyte populations of these depots. *Tcf21* mRNA content was lower in GON from females compared to males and mPVAT from both sexes. No differences in the expression of brite adipocyte identity markers, *Tbx1* and *Tmem26,* were observed across sites.

### SVF and APC proliferative and adipogenic capacity

**Figure 4:**
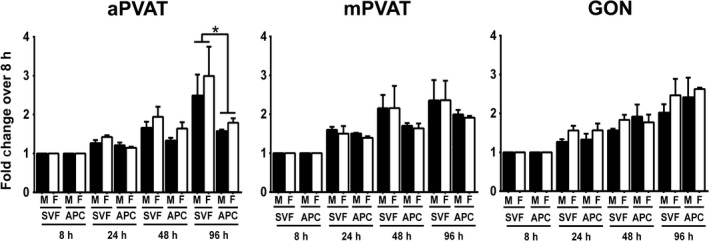
In vitro proliferation of stromal vascular fraction (SVF) cells and adipocyte progenitor cells (APC) is affected by anatomical location but not sex. SVF cells and APC were harvested from aortic (aPVAT) and mesenteric (mPVAT) perivascular adipose tissues and perigonadal (GON) fat depot from 10‐week‐old males (*n* = 4) and females (*n* = 6). Cell expansion was measured using a DNA quantification assay (CyQuant^®^) at 8, 24, 48, and 96 h post seeding. Data are expressed as the fold increase over 8 h ± SEM. Significant differences indicated by * (*P* < 0.05).

Adipose tissue expansion is dependent, at least in part, on the proliferative capacity of preadipocytes and the adipogenic potential of these adipocyte precursors. Importantly, these characteristics are maintained in vitro (Tchkonia et al. 2006). Male and female SVF and APC collected from aPVAT, mPVAT, and GON were isolated and their in vitro proliferation was evaluated at 8, 24, 48, and 96 h after plating using a quantitative DNA assay (CyQuant^®^). There were no sex or site differences in SVF expansion rate at any time point (Fig. 4). However, APC from aPVAT had a slower proliferation rate by 96 h compared to SVF cells from the same site (Fig. 4).

**Figure 5:**
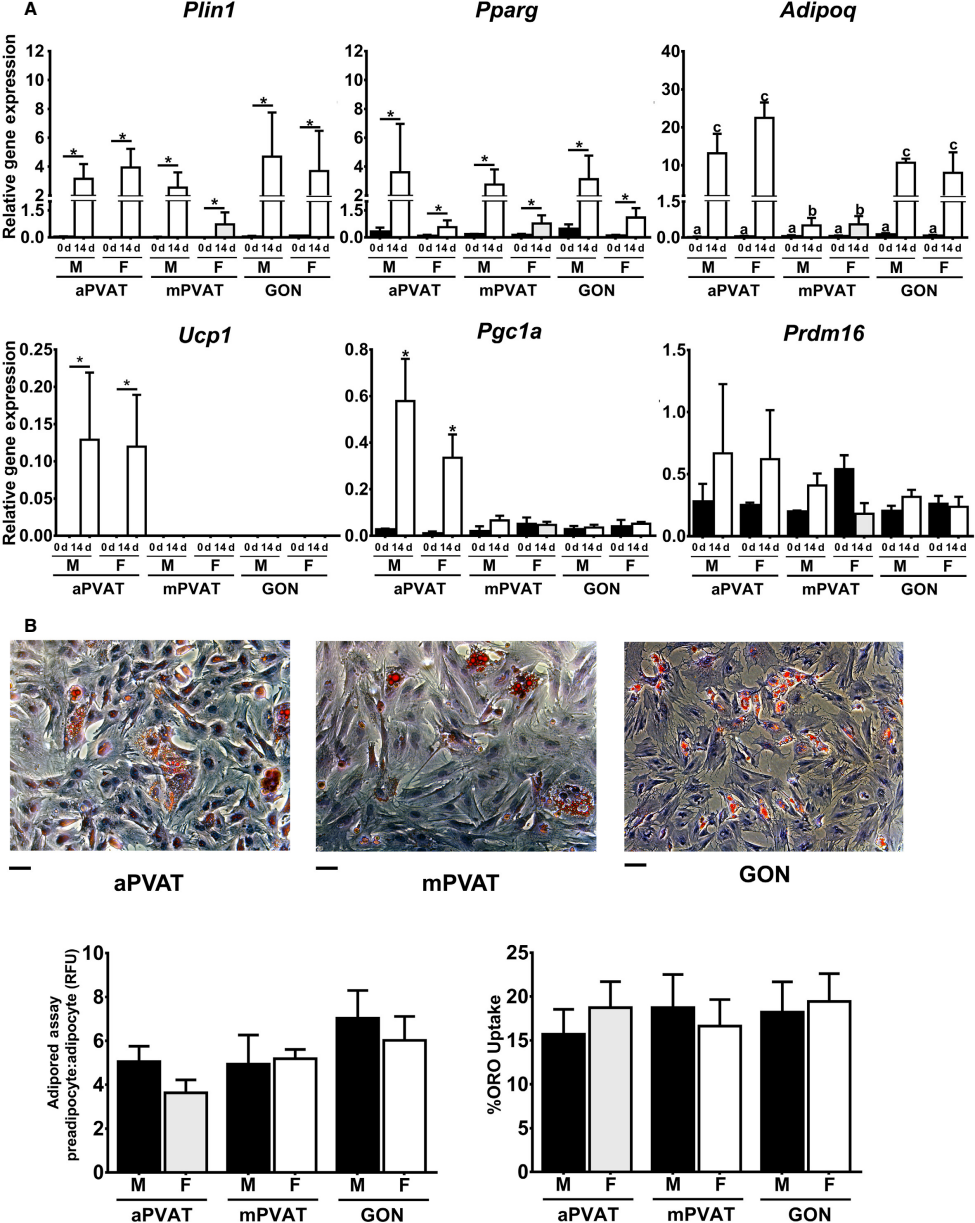
Adipose signature genes expression in stromal vascular fraction (SVF) cells from perivascular adipose tissues is influenced by sex and anatomical location. (A) Expression of adipogenic and brown adipocyte‐related genes in SVF from aortic (aPVAT) and mesenteric (mPVAT) perivascular adipose tissues and perigonadal (GON) fat depot from 10‐week‐old males and females (*n* = 4). Gene expression was evaluated by RT qPCR and normalized to *Rps29*. Data are means ± SEM. Significant differences are indicated by * (*P* < 0.05). (B) Representative high‐magnification images of SVF from aPVAT, mPVAT, and GON stained with hematoxylin and Oil Red O (ORO). Scale bars = 100 *μ*m. Lipid accumulation in SVF as measured by the AdipoRed^™^ assay and ORO uptake. AdipoRed^™^ data are expressed as the ratio preadipocyte: adipocyte relative fluorescence units (RFU).

Confluent SVF cells were induced to differentiate using a standard induction protocol for 14 days (Zheng et al. 2013). The expression of adipocyte‐specific genes including: perilipin 1 (*Plin1*), *Pparg*, and *Adipoq* were evaluated after induction. The expression of these genes was significantly increased in induced SVF cells compared to preconfluent SVF (Fig. 5A). There was no effect of sex on the gene expression of adipogenic genes in induced SVF. However, induced SVF from male and female mPVAT had a lower expression of *Adipoq* compared to other sites. Total differentiation efficiency, as evaluated by intracellular lipid accumulation using the Oil Red O assay, was 17.91% ± 0.59 and was not affected by sex or site (Fig. 5B). Reflecting Oil Red O results, AdipoRed^™^ assay lipid content ratio (RFU adipocyte: RFU preadipocytes) was 5.31 ± 0.46 across sexes and sites. For comparison, 3T3‐L1 adipocytes have lipid accumulation ratios greater than 10.

**Figure 6:**
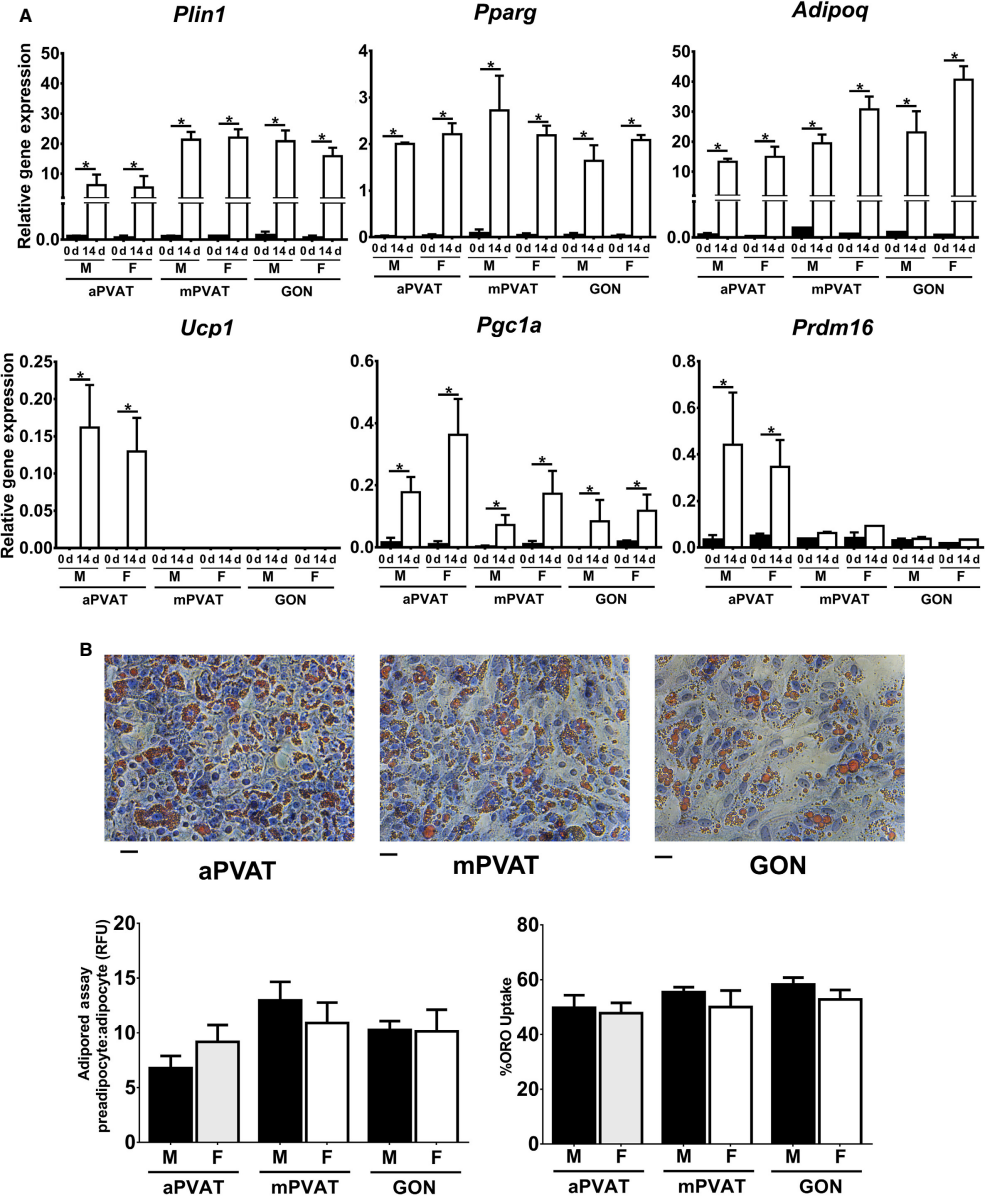
Adipose signature genes expression in adipocyte progenitor cells (APC) from perivascular adipose tissues is influenced by sex and anatomical location. (A) Expression of adipogenic and brown adipocyte‐related genes in APC from aortic (aPVAT) and mesenteric (mPVAT) perivascular adipose tissues and perigonadal (GON) fat depot from 10‐week‐old males and females (*n* = 3). Cells were induced postconfluency after exposure for 48 h to bone morphogenic protein‐4 using a standard adipogenic protocol for 14 days. Gene expression was evaluated by RT qPCR and normalized to *Rps29*. Data are means ± SEM. Significant differences are indicated by * (*P* < 0.05). (B) Representative high‐magnification images of SVF from aPVAT, mPVAT, and GON stained with hematoxylin and Oil Red O (ORO). Scale bars = 100 *μ*m. Lipid accumulation in APC as measured by the AdipoRed^™^ assay and ORO uptake. AdipoRed^™^ data are expressed as the ratio preadipocyte: adipocyte relative fluorescence units (RFU).

When confluent APC were induced using the same protocol (Zheng et al. 2013), adipogenesis efficiency was similar to that of the SVF. However, stimulation of APC with bone morphogenic protein‐4 for 48 h prior to the standard induction protocol for 14 days, as described in Macotela et al. (2012), induced a strong expression of adipogenic genes *Plin 1*,* Pparg*, and *Adipoq* independently of sex or site (Fig. 6A). The expression of brown phenotype gene *Ucp1* was only detected in differentiated APC of male and female aPVAT. *Pgc1α* was expressed in differentiated APC from both sexes and all sites (*P *<* *0.05). *Prdm16* was significantly higher in differentiated APC from aPVAT compared to mPVAT and GON in both females and males. Differentiated APC exhibited higher induction rates compared to SVF induced with standard protocols and this was reflected in greater lipid accumulation as evaluated by AdipoRed^™^ and Oil Red O staining (Fig. 6B).

## Discussion

The expansion of aPVAT and mPVAT is directly linked with the development of CVD (Brinkley et al. 2014; Fitzgibbons and Czech 2014; Aghamohammadzadeh et al. 2015). Sex‐ and site‐related differences in the enlargement of these PVATs may be related to the distribution and adipogenic capacity of specific APC populations that are currently unknown. In this study, we demonstrate that aPVAT and mPVAT exhibit a unique distribution of APC and gene expression signatures that reflect sexual dimorphism and differences due to anatomical location.

### APC markers in PVAT: sex and site differences

Our results reveal for the first time that females have a significantly lower number of CD34^+^ SVF cells in PVATs and GON compared to males. This finding may partially explain the sexual dimorphism observed in adipose tissue partitioning across species. In humans, males accumulate more visceral fat during development, aging and obesity even after adjusting for BMI (reviewed by Tchernof and Després 2013). CD34 is a mesenchymal cell marker which is consistently expressed in APC (Cawthorn et al. 2012). This protein is an important mediator of cellular trafficking and cellular morphogenesis and at least 75% of CD34^+^ SVF cells are committed to become APC (Nielsen and McNagny 2008; Church et al. 2014). Hyperplasia in adipose tissues is dependent on the number of CD34^+^ cells. This was demonstrated in fat tissue transplantation studies where adipose tissue graft survival was dependent on CD34^+^ cell abundance (Philips et al. 2013). Therefore, a higher number of CD34^+^ in male PVAT may explain, in part, its higher expansion capacity compared to female PVAT.

A second important observation in PVATs’ APC distribution profile was the lower aPVAT cell surface expression of PDGFR*α* alone or coexpressed with CD34 and CD44. Using lineage tracing techniques, APC (CD34^+^, CD44^+^, PDGFR*α* ^+^) were found to be highly proliferative and with the potential to become white or brown adipocytes in different fat depots (Lee et al. 2012, 2013). During diet‐induced obesity, these cells proliferate and undergo adipogenesis becoming unilocular adipocytes in GON; therefore, driving the hyperplasia response to a high caloric intake (Lee et al. 2012). Reduced populations of PDGFR*α* ^+^ cells in aPVAT SVF may implicate a limited capacity for expansion during obesity that could be further restricted by the observed resistance of these cells to differentiate (in vitro) in the presence of a standard adipogenic protocol. Restricted adipogenic potential is also observed in human coronary PVAT SVF (Chatterjee et al. 2009); however, it is not presently known if this limited expansion capacity is associated with a low number of APC in coronary PVAT.

### Sex and vessel location influence PVAT adipocyte morphology and signature genes expression

Previous studies in humans and rodents demonstrated that PVAT adipocytes are smaller than those localized in nonperivascular sites such as GON (Chatterjee et al. 2009; Kranendonk et al. 2015). The present work also confirmed that adipocytes from PVATs in the abdominal cavity are larger than those localized in the thorax (e.g., aPVAT) as described by Padilla and colleagues (Padilla et al. 2013). A novel finding in our study was the differences in PVAT adipocyte area distribution between males and females. Larger adipocyte size was reported in non‐PVAT visceral depots from men compared to women (Fried and Kral 1987; Tchoukalova et al. 2010). Variations in adipocyte size influence adipose tissue inflammatory responses as larger fat cells are associated with enhanced adipose tissue remodeling and immune cell infiltration in omental and abdominal aorta PVAT (Chatterjee et al. 2009; White and Tchoukalova 2014). It is presently unknown if larger adipocyte sizes in male PVATs may be associated with higher risk for vascular dysfunction compared to females.

Despite similar histological morphology, aPVAT does not share the same developmental origin as BAT. This was demonstrated in our study by the lack of *Zic1* expression in aPVAT. This gene encodes zinc finger proteins that are essential for early skeletal muscle and BAT development in mammals (Himeda et al. 2013; Mathur et al. 2013). Similar to our results, de Jong and colleagues observed no expression of this developmental gene in murine aPVAT (de Jong et al. 2015). Genetic tracing further confirmed dissimilar gene expression patterns between BAT and aPVAT by demonstrating that the latter is not derived from the well characterized BAT lineages My5 or Pax3 (Sanchez‐Gurmaches and Guertin 2014). Given the recent identification of clinically relevant brown adipose depots surrounding thoracic aorta and coronary arteries in men and women of different ages (Wei et al. 2015), a complete characterization of aPVAT development and expansion during aging and obesity is warranted.

The developmental origins of mPVAT are similar to those of GON, a nonperivascular white fat depot with similar histological characteristics. In our study, both adipose depots exhibited analogous expression of the white adipocyte signature gene *Tcf21* and the brite adipocyte‐related genes *Tbx1* and *Tmem26*. In line with this finding, recent lineage tracing experiments demonstrated that mesenteric fat and GON share the same mesothelial origin and may have similar mechanisms for metabolic regulation (Chau et al. 2014). Despite these similarities, mPVAT adipocytes exhibited smaller cellular size than GON. In mice and humans, GON adipocytes are also larger than mesenteric fat cells (Caesar et al. 2010). Because of their smaller size, mPVAT adipocytes have limited hypertrophy compared to fat cells from GON, omental, and subcutaneous depots (Fang et al. 2015). As a consequence, once mPVAT adipocytes reach their hypertrophy threshold, this PVAT may be more susceptible to inflammatory responses compared to other white adipose depots.

A second finding that highlights the differences between mPVAT and GON were the contrasting patterns in *Adipoq* expression observed in our study. In males, GON had higher *Adipoq* transcription levels than mPVAT from both sexes. Similar findings were reported in male mice before and after treatment with a proadipogenic PPAR*γ* agonist (Vernochet et al. 2009). Female GON was also found to have lower *Adipoq* expression compared to male GON and retroperitoneal and inguinal white adipose depots from male and female Wistar rats after 15 days of cafeteria diet feeding (Ribot et al. 2008). *Adipoq* expression in cultured SVF cells reflected whole tissue findings; however, in cultured APC, the transcription of the adiponectin gene was not affected by sex or anatomical site. This may be related to the modulation of adiponectin expression by nonadipogenic cells within the SVF. Previously Hino et al. (2012) reported that bone morphogenetic protein‐3b reduced adipogenesis efficiency and adiponectin gene and protein expression during in vitro culture of SVF cells from mesenteric adipose tissues. Since adiponectin is recognized as an important anticontractile agent specially in small arteries (Lynch et al. 2013), such as those surrounded by mPVAT, the role of specific populations of APC and other SVF components in the secretion of this adipokine during hypertension or other CVD will require further characterization. Similar to *Adipoq*, the expression of *Tcf21* in GON from female rats was lower compared to male GON and mPVAT from both sexes. *Tcf21* is a regulator of cell differentiation in male gonads and smooth muscle of coronary arteries (Bhandari et al. 2011; Nurnberg et al. 2015); however, its role on the divergent adipogenesis responses between males and females remains to be elucidated.

### Limitations

It is necessary to mention that our study only focused on a specific subpopulation of APC. Other research groups have identified additional committed progenitors with diverse cell surface marker profiles (Zimmerlin et al. 2010; Gupta Rana et al. 2012; Sanchez‐Gurmaches and Guertin 2014), although not in PVAT, that may have different adipogenic and hypertrophy responses during development, aging, and obesity. Given the diverse characteristics of PVAT adipocyte populations, it is also possible that APC with a specific cell surface marker profile may have different phenotypes depending on anatomical location. A technical limitation of this study is that the analysis of APC populations was based solely on flow cytometry and did not evaluate in vivo adipogenic potential since the availability of rat models for cellular lineage tracing is currently very limited. This study was an initial foray into the effects of sex and anatomical site on APC population distribution to provide specific adipogenic and vasoactive targets for future functional studies in the context of high fat diet feeding and the absence of gonadal hormones. Finally, it is important to note that this study focused exclusively on mPVAT (fat surrounding mesenteric resistance arteries), whereas other studies do not distinguish collection sites and harvest the entire mesenteric fat (Tchkonia et al. 2006; Caesar et al. 2010; van Beek et al. 2015). An objective distinction between perivascular and nonperivascular fat other than anatomical location remains to be established.

## Conclusions and Perspectives

Our study presents evidence that APC in PVAT exhibit unique gene expression signatures and have specific anatomical site distribution and sexual dimorphism in the expression of CD34. Future work should determine if these unique characteristics may explain in part the apparent aPVAT resistance to expansion in early stages of obesity and if higher number of APC in males explicate its rapid expansion compared to females.

## Conflict of Interest

No conflicts of interest, financial or otherwise, are declared by the authors.
